# Anti-aging and Sunscreens: Paradigm Shift in Cosmetics

**DOI:** 10.15171/apb.2019.042

**Published:** 2019-08-01

**Authors:** Shreya Shanbhag, Akshatha Nayak, Reema Narayan, Usha Yogendra Nayak

**Affiliations:** Department of Pharmaceutics, Manipal College of Pharmaceutical Sciences, Manipal Academy of Higher Education, Manipal 576 104, India.

**Keywords:** Aging, Anti-aging, Nanoparticles, Photoprotection, Sunscreen, Sun protection factor (SPF)

## Abstract

Skin, being one of the vital organs and a protective barrier needs to be pampered and taken care of from early childhood. It is the most visible and the widest exposed organ and by far reflects the general health condition and the aging process in humans. Both intrinsic and extrinsic factors contribute to this complex biological process of skin aging. In recent times, skin health and its beauty is perceived as an indicator of one’s health which has resulted in an increasing demand for anti-aging products. Exposure to UV radiation is considered to be one of the factors responsible for aging termed as photoaging. In this review, we have discussed the various factors which may accelerate the process of skin aging. Various approaches and strategies to delay the process of skin aging have been emphasized upon. The patents filed in the area of anti-aging and sunscreen products have also been reviewed to gain an insight into the new formulations which have been developed as an anti-aging product. There has been a tremendous rise in the cosmetic and cosmeceuticals market with products having a dual activity of anti-aging and sun protection. Research is constantly on the rise to ensure the safety of these products. Alternatives to the current topical application of sunscreen are being considered to overcome the drawback of reapplication of the sunscreen often which can be a boon to the cosmeceutical market.

## Introduction

Skin is a protective layer of the body of any animal including humans. As the age progresses, certain changes occur in the skin which are influenced by certain extrinsic and intrinsic factors.^1^ The changes in the skin are among the most visible signs of aging which include wrinkles, sagging skin, age spots and dryness, and also loss in the fat making the skin lose its natural smoothness. The skin is mainly composed of three layers, the outer part epidermis, middle part dermis, and the innermost subcutaneous layer. As a person ages, the epidermis slowly thins even though the number of cell layers remains same.^2^ The inherent repairing ability of skin gradually reduces as a person ages which may be due to infections and pressure ulcers. In addition, the number of melanocytes decreases, aging skin becomes thinner, paler and clear with large pigment spots, age spots or liver spots. All these signs necessitates the need for the anti-aging treatment.^3^ As we age, our body produces less collagen and elastin which plumps our skin and makes it lose its elasticity respectively. By the use of anti-aging products or treatment, either the collagen production can be boosted, or its natural loss can be slowed down.^4^ Anti-aging treatments are also necessary to reduce fine lines, wrinkle, acne and it also helps in making the skin firm.

Although sun-exposure is very essential for the synthesis of vitamin D, exposure to harmful UV rays results in premature aging, initiation of the reactive oxygen species generation, skin cancer, and degradation of extracellular matrix components *viz,* collagen type I, fibronectin, elastin and proteoglycans induced by mitogen-activated protein kinase signaling pathway upregulation. Application of sunscreens to the exposed parts of the skin may help in protecting the skin from harmful UV rays. Sunscreens are the products combining several ingredients which protect the skin by absorbing, blocking or scattering UV radiation.^5^ Two types of agents protect the skin from sunlight, one which reflects the UV rays and the other which absorbs the UV rays. Reflectors are the substances which when present on the surface of the skin reflects the UV rays thus preventing them from entering the skin. Absorbers absorb the sunlight, and they are active against a specific spectrum of sunlight. So these can be used individually or in combination to act as a sunscreen. The sunscreen with a minimum sun protection factor of 15 (SPF15) should be used to obtain the maximum benefit.^6^

In the present review, we have discussed on the aging mechanisms and various strategies to combat the various signs of aging. We have also touched upon the advances in the field of nanotechnology and its importance for the delivery of cosmeceuticals having anti-aging and sun protecting effect.

## Skin aging and its mechanism

**Figure 1:**
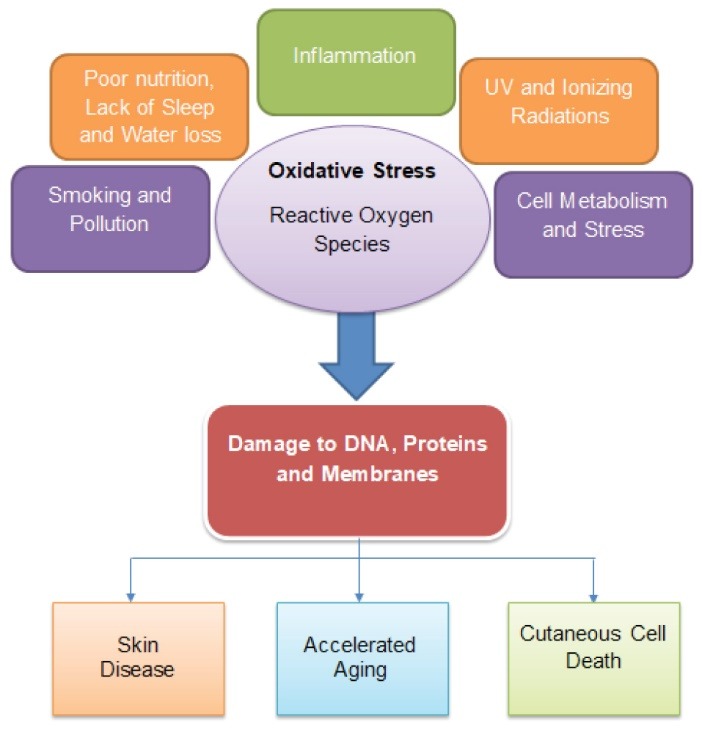
Causes of aging.

Skin aging is a complex biological process involving a blend of multiple components. Although the underlying mechanism of skin aging is not yet completely understood, multiple pathways were illustrated which were speculated to be responsible for skin aging namely changes in DNA repair and stability, mitochondrial function, cell cycle and apoptosis, ubiquitin-induced proteolysis, and cellular metabolism. The most vital factor responsible for skin aging may be a decline in the physiological hormone.^7^ UV radiations contribute about 80% in the skin disease development including skin aging and skin cancer.^8^ Thus UV radiation is a causative factor for skin aging.^9^ Repeated exposure to UV increases the degradation of collagen and alters the synthesis of new collagen accompanied by alterations in elastin fibers. The absence of both collagen and elastin in the skin leads to loss of its flexibility and strength.^10^ In addition, the skin also loses the ability to repair itself. The different causes of aging are depicted in Figure 1.

## Factors responsible for skin aging

Most of the changes occurring in the skin may be attributed to a mix of both, endogenous/intrinsic and exogenous/extrinsic factors. Intrinsic aging causes changes in the epithelial cell layer whereas extrinsic aging causes abnormal accumulation of elastic tissue in the dermis.

### Intrinsic factors

It is an endogenous mechanism of aging due to hormonal changes, genetic factors and cellular mechanisms, and several other mechanisms.

*Free radical mechanism*: To maintain equilibrium in our body there is a constant generation and removal of the free radicals. An imbalance in this process results in the formation of excessive free radicals which are toxic to the body and cause aging.^11^

In the process of intrinsic aging, these free radicals are formed by oxidative cellular metabolism. The free radicals produced in the process, are removed by anti-oxidative mechanisms, but as the age progresses, there is a decrease in the anti-oxidative mechanisms and eventually excessive free radicals in our body which leads to cellular aging.^12^

*Hormonal mechanisms*: The skin aging takes place by certain modification in growth factors and hormonal activity. The decline in several hormones in our body such as estrogen, testosterone, dehydroepiandrosterone and its sulfate ester and also melatonin, insulin, cortisol, thyroxine, and growth hormone can deteriorate several skin functions.^13^ In postmenopausal women, there is a decline in the estrogen levels which results in several aging signs such as dryness, wrinkles, loss of elasticity, collagen breakdown and epidermal atrophy.^12^

*Mitochondrial DNA damage*: Mitochondria consume oxygen and produce energy, and as a result, there is a continuous production of reactive oxygen species. These reactive oxygen species causes oxidative stress after exhaustion of cellular defense mechanisms and they also cause further mutation of mitochondrial DNA. These mitochondrial DNA cause high mutation rates because of inefficient recognition and repair mechanism.^12^ This damaged mitochondrial DNA produces less energy which affects the energy supply to the cells which in turn lead to cellular dysfunction. The damaged mitochondria undergo degeneration, rupture, leakage which are the prime reasons for aging.^11^

*Role of telomere*: Telomere protects the chromosomes from degradation and also prevents cellular DNA damage. Due to shortening of the telomeres, the t-loop configuration is disrupted which initiates DNA damage response, apoptosis, senescence or cell cycle arrest. Hence the shortening of the telomeres is responsible for intrinsic aging and photoaging.^14^

### Extrinsic factors

*Smoking*: Smoking damages the collagen and elastic fibers in the dermis which makes the skin more slack, hardened and less elastic. Nicotine, carbon monoxide and other toxic substances produced during smoking result in vasoconstrictive and hypoxic effects on the skin. These contribute to premature skin aging.^15^

*Ultraviolet (UV) radiations*: About 80% of the facial aging is attributed to sun exposure. Photo damaged skin contributes for loss of skin elasticity, skin roughness and dryness, irregular pigmentation and deep wrinkling.^16^

*Life style*: It also has a major impact on aging. Lack of exercise, alcohol consumption, unhealthy diet, pollution, stress contributes to aging. Certain lifestyle factors cause an increase or decrease in the rate of telomere shortening. Ensuring a healthy lifestyle is important in reducing the telomere shortening and thus slowing down the aging process.^17^

## Oxidative stress in aging

Oxidative stress has been linked to age-related loss of elasticity of the skin. The main cause of oxidative stress is the presence of excessive reactive oxygen species. In normal conditions, they are produced in our body during oxidative phosphorylation in mitochondria; however, these are inactivated by cellular antioxidant defense mechanism. Oxidative stress triggers cellular damage pathways and causes senescence of cells which may lead to photoaging. Exposure to UV rays specifically UVA rays can alter the levels of reactive oxygen species and the protective antioxidant enzymes such as manganese superoxide dismutase, copper/zinc superoxide dismutase and catalases.^18^ The reactive oxygen species generated not only cause skin aging but also acts as a toxic agent in developing cancer, inflammation, cardiovascular diseases and numerous skin diseases.^19^ UVB radiations also initiate photochemical production of reactive oxygen species mainly superoxide anion, hydrogen peroxide, hydroxyl radical and singlet oxygen. To reduce the effect of this damaging solar radiation, sunscreens can be used which protects the skin from harmful sun exposure. Oxidative stress can also be reduced by dietary changes and special nutrients, and thereby reduce skin damage process.^8^

## Signs and mechanism of skin aging

Wrinkles and sagging of the skin are the two major signs of skin aging. The underlying mechanism is still poorly understood. The wrinkled skin has an accumulation of altered elastic fibers and degradation of collagen bundles in the dermis. Enhanced elastase activity in dermal fibroblasts is mainly associated with UVB wrinkling mechanism. The activation of cytokine expression in epidermal keratinocytes by UVB irradiation causes the secretion of IL-1α and GM-CSF which penetrates into the dermis. This stimulates the expression of skin fibroblast elastase which cleaves the elastase fibers leading to a loss in its configuration thereby leading to reduced skin elasticity leading to wrinkle formation.^20^

## Prevention of aging

Different approaches are available for the prevention and delay of skin aging. Extrinsic aging is largely preventable than intrinsic aging. Photoprotection helps to prevent skin aging which includes the use of sunscreens, protective clothing and sunglasses, avoiding sun’s harmful radiation thereby reducing the progression of skin aging. Antioxidants also help in the prevention and treatment of intrinsic and extrinsic skin aging by acting as free radical scavengers thereby preventing the cells from damage.^21^ Treatment with antioxidants such as ascorbic acid, polyphenols, tocopherols, and other natural substances helps to develop resistance to oxidative stress and slows down the process of skin aging.^22^ Phytochemicals such as resveratrol, quercetin, green tea extract have also been reported to be effective in reducing the progression of the aging process.^23^ Apart from these, topical treatment with cell regulators like vitamin A derivatives, polyphenols, and botanicals also helps in preventing aging. These act on collagen metabolism thereby stimulating the production of collagen and elastic fibers.

## Strategies for anti-aging

The proportion of the aged population is gradually on the rise owing to developments in healthcare and improvement in lifestyle, particularly in developed countries. With aging, some of the body functions get affected leading to a variety of diseases like a chronic coronary disease, hypertension, and diabetes.^24^ Increased production of oxygen-derived free radicals plays a vital role in the aging process.^25^ Aging is a process that affects all cells, tissues, organs, diminishing homeostasis and increasing organism vulnerability.^26^ Premature photoaged skin leads to the thickened epidermis, deep wrinkles, discoloration, roughness, and dullness.^27^ Further loss of skin elasticity leads to a phenomenon called sagging. This, in turn, leads to less effective desquamation and slower wound healing in older people. It is a fact that skin beauty is perceived as an important indicator signifying the overall well-being of an individual. Hence several anti-aging strategies are developed. Some of them are skin care, use of moisturizing preparations, botulinum toxin, hormone replacement therapy, antioxidants, photoprotection, and anti-wrinkling treatment.

As age progresses, the skin tends to become dry and scaly, especially in the elderly. So it becomes important to use a barrier to preserve this vital layer. Protection against dehydration, preventing the penetration of irritants, microorganisms, allergens, radiation, and protection against the reactive oxygen species requires a healthy and functioning skin barrier. Penetration via the skin can be regulated to allow selective penetration of substances which helps in skin regeneration, maintaining smoothness and elasticity.^1^ Degradation of primary structural components, i.e. elastin and collagen results in the formation of wrinkles. So, care should be taken by keeping the skin subtle and moist; this will help the skin look radiant and younger. Personal care products are available from many sources for this purpose.^28^ Some of the practices that can improve the skin conditions are drinking more water, eating healthier foods, reducing stress, using sunblock and exercising more. Another approach is the use of topical or systemic antioxidants which helps in the prevention of wrinkles by reducing inflammation. Some of the skin care approaches are discussed below.

### Hormone replacement therapy

Months after menopause, females experience a sudden onset of the symptoms of skin aging like dryness and a decrease in skin elasticity. The decrease in estrogen might thus reduce the skin functions which are in control of estrogen.^29^ Studies have shown that hypo-estrogenism affects the skin collagen. Skin collagen content varies according to age, and it declines in the years following menopause. Increase in skin collagen was found in people who underwent hormone replacement therapy.^30^ However, the evidence for the same is limited and hence remains to be controversial. In one of the studies, an increase in the levels of procollagen I and III messenger RNA and collagen I protein was observed in the sun protected aged hip skin in subjects treated with topical estradiol. However, surprisingly, no beneficial effects were observed when applied on photoaged forearm or face skin.^31^

### Botulinum toxin

Botulinum toxin (BTX) does not stop the aging process, but these BTX injections help in slowing down of the visible aging processes such as facial lines and wrinkles. Botulinum toxin type A (BTX-A) injections are the most effective cosmetic procedures for reducing the appearance of facial lines caused by facial muscle contraction. BTX-A is one of the most effective approaches for wrinkles which form because of muscular contraction. It works by selectively blocking the release of acetylcholine at the neuromuscular junction by binding to presynaptic nerve terminal and thereby it prevents muscle contraction. After this injection, improvements were found in about 1-14 days. BTX label recommends its use only for treating glabellar lines among adults below 65 years of age, however, it is still being used for different cosmetic purposes.^32^ BTX-A has the advantage that it does not cross the blood-brain barrier neither does it permeate through the skin. The dosing largely depends on the area, muscle mass and gender.^1^ Elders above 65 years of age are likely to have weaker facial muscle, less elastic skin and are hence not expected to respond well to this therapy.^33^ The use of botulinum toxin type A has rapidly increased over the years, and it has occupied a major portion in the cosmetic care market. Its use has become synonymous with wrinkle reduction and has formed the basis for the identification and synthesis of many other peptides which may have similar activity and are relatively safe as compared to botulinum toxins.^34,35^

### Anti-wrinkling

Wrinkles are mainly caused due to lack of elastic feature of the skin. A specific skin fibroblast elastase inhibitor can be used to prevent UVB induced wrinkle formation and thus help to maintain the linear configuration of elastic fibers and skin elasticity.^20^ Hyaluronic acid and botulinum toxin have also been widely used as anti-wrinkling agents.^36^

### Moisturizing preparations

Hyaluronic acid plays a principal role in the preservation of hydration and elasticity of the skin. Sericin gel also has moisturizing effect when applied on the skin. This may be attributed to the hydration of epidermal cell and an increase in hydroxyproline level. This aids in skin care and anti-aging effect of sericin.^37^

### Antioxidants and photoprotection

The primary strategy for prevention of photoaging is photoprotection, and the secondary treatment is by the use of exogenous antioxidants and other compounds that cannot be synthesized in our body. Polyphenols, a novel set of compounds have garnered widespread interests as effective anti-aging compounds possessing significant antioxidant properties. They prevent the formation of reactive oxygen species triggered by exogenous (UV radiations) and endogenous agents thereby inhibiting oxidative skin damage.^8^ Vitamin B3, C, and E which shows sufficient penetration into the skin form the most important antioxidant substances. Several other compounds like alpha lipoic acids, alpha hydroxyl acids in conjunction with vitamins have proved to be beneficial as antioxidants.^38,39^ Regular use of antioxidants such as vitamin A, C, E, etc. in the normal diet, may reduce the risk of UV induced skin damage. Antioxidants prevent oxidative stress and enhance DNA repair. Defense mechanisms by the cellular antioxidants help to prevent the damage caused by the oxidizing components of UV radiations.^8^ Antioxidants react with the superoxides and suppress the skin diseases caused by reactive oxygen species.^19^ The different types of free radical scavengers are superoxide dismutase, coenzyme Q10, vitamin E, vitamin C, zinc sulfate, green tea, ferulic acid, idebenone, polyphenols, and carotenoids.^11^ Several photoprotective agents are nothing but protective clothing, umbrellas, trees which acts as sunblock. Sunscreen which has several inorganic and organic filters also helps in photoprotection.^40^

## Sunscreens

Sunscreen agents protect the skin by minimizing the damaging effects of harmful UV radiations from the sun. About 90 % of cases of skin cancer are associated with exposure to the sun’s harmful UV rays. UV radiation that irradiates the earth is absorbed by the ozone layer. Consequently, UVA and UVB reach the earth surface. UVA reaches the earth surface and contributes to premature skin aging and skin cancer, while, UVB causes sunburn. Of the UV radiations reaching the earth, UVB is responsible for most of the deleterious effects of solar exposure, but the damaging role of UVA has been greatly documented. Hence, new generation sunscreen which protects a whole range of UV radiation is recommended.^41^ Sunscreens are strongly recommended by many healthcare practitioners to minimize the harmful effects of UV rays on our skin.^42^ The most important reason behind the use of sunscreen is that it shields us from harmful UV rays, prevents premature aging, tanning, and sunburns, lowers blotchiness on the face, improves the health of the skin, and lowers the incidence of skin cancer.^43^ UVA rays upregulates the expression of matrix metalloproteinase which degrades elastin and collagen, the two major components which maintain the rigidity of skin. Failure to protect from the harmful effects of UVA radiation may lead to loss of elasticity and lead to wrinkling of skin.^44^

### Sunscreen ingredients/sunscreen filters

The efficiency of the sunscreen depends on the type of UV filter which may be an organic or an inorganic UV filter.^45^ The sunscreen ingredients are broadly classified into two types *viz*, chemical or organic and physical or inorganic.^46^ Based on their mechanism of action, sunscreens are also traditionally divided into inorganic (physical) blockers and organic (chemical) absorbers.

#### Organic filters

Organic filters constitute those substances which are capable of absorbing UV radiation of a specific range of wavelength based on their chemical structure.^47^ Broad spectrum filters used in sunscreen have a higher level of absorption. The organic filter molecules absorb UV energy and transform to higher energy state from the ground state.^41^ Excess energy is released via isomerization and heat release resulting in the emission of higher wavelengths or relaxation.^47^ If these filter molecules are photostable, they will deactivate to its ground state from the excited state with the release of absorbed energy in the form of heat.^41^ Organic sunscreens are classified as derivatives of anthranilates, benzophenones, camphor, cinnamates, dibenzoylmethanes, para-aminobenzoates, and salicylates.^48^ The organic filters can be classified into:

*a) Photostable filters*: These are the molecules which dissipate the absorbed energy to the surroundings in the form of heat. These filters efficiently absorb UV energy subsequently.

*b) Photounstable filters*: These are the molecules which undergo degradation or change in their chemical structure on the absorption of UV radiation. Hence, these cannot absorb UV energy on subsequent exposure.

*c) Photoreactive filters*: These molecules jump to their excited state on irradiation with UV. Once in their excited state, they interact with other molecules in their surrounding including the ingredients of sunscreen, skin lipids and proteins. This interaction results in the production of reactive species, resulting in untoward biological effects.^49^

#### Inorganic filters

Inorganic filters include zinc oxide, titanium dioxide, iron oxide, red veterinary petrolatum, kaolin, and calamine. These filters are said to block UVB/UVA sunlight through scattering and reflection. Two major characteristics that determine the ability of minerals to act as physical filters are scattering/absorption property and cosmetic acceptability.^50^ They include metal oxides like titanium dioxide or zinc oxide which is used in combination with organic filters. Inorganic filters protect the skin by reflecting and diffusing UV radiations. The larger metal oxide particles tend to diffuse the light from the visible range of the spectrum leaving an undesirable whitish appearance on the skin.^41^ The whitening effect and opaque nature are some disadvantages of these filters, which can be minimized to some extent by the use of ultrafine particles.^49^

### Sunscreens and sunblocks

Sunblocks are the products which protect against UVB rays while sunscreen protects against UVA rays. Sunblock comes under the physical category of protective lotion, containing both non-organic and organic constituents that get deposited on the surface of the skin when applied, shielding the skin from harmful UVB rays. These block the rays from penetrating the skin. Some of the ingredients found in sunblock are octocrylene, octyl salicylate, and octyl methoxycinnamate. The chemical type of protective lotions can be broadly referred to as sunscreens. This lotion permeates through the skin and absorbs the UVA rays before they can reach and damage the dermal layer. Zinc oxide and titanium dioxide constitute the sunscreen. Ecamsule is one of the ingredients present in sunscreen. This compound shields the skin from the photoaging UVA rays. A formulation which constitutes both sunscreen and sunblock ingredients serves as a protective agent against both UVB and UVA rays, and a combination of these two is more efficient in the protection of the skin than using them alone.^51^

When sunscreen is applied as recommended, it tends to reduce only 55% of the free radical formation. However, antioxidants in sunscreen are capable of reducing greater amount of free radicals than the use of sunscreen alone.^52^ Formulation of sunscreen containing antioxidant faces certain challenges such as ensuring the stability of antioxidants in the final formulation. A challenging task in the formulation of these products is that the antioxidant needs to penetrate into the epidermis whereas the UV filters need to remain on the surface of the skin to exert its effect. Hence, a careful modulation of the formulation is essential to maintain a balance between both the properties of the product. Even though our body has an internal antioxidant defense mechanism to neutralize reactive oxygen species, this defense mechanism might get depleted during excessive oxidative stress. Here the topical administration of the antioxidants might help to reduce the reactive oxygen species. So these antioxidants provide additional benefits to the sunscreen formulations and skin care products.^53^

## Anti-aging and sunscreens

Broad spectrum sunscreens protect the skin from aging as well as harmful UV rays. Some products also claim protection against free radicals.^54^ Some of the products that show both properties are listed in Table 1.

**Table 1:** List of some marketed products with both anti-aging and sunscreen properties

**Product name**	**SPF**	**Claims**	**References**
Clinique	30	Mineral sunscreen fluid for face, high-quality sun care product	55
Kiehl’s activated sun protector	50	Water resistant facial sun cream that protects from UVB	56
La Roche Posay Anthelios XL	50	The ultra-light fluid that’s scent-free and won’t clog pores	57
Nivea	50	An anti-age face cream that helps reducing wrinkles and fine lines and also highly moisturizing and protects from UV rays	58
Clarins	15, 30	Sun wrinkle control cream for face. It provides sun protection and contains anti-aging properties	59
Ultrasun face	30	Anti-aging formula, this sunscreen fights against free radicals	60
Alpha H	50	It is a multipurpose beauty product. It is a moisturizer which protects the skin from UV rays, free radical damage and photo aging	61
Frezyderm	30	It provides protection against UVB and it also helps prevent photoaging	62

There has been a lot of research conducted on anti-aging and sunscreen products to enhance its efficacy, stability, safety and be more favorable to consumers. Some of the literatures available are discussed below.

Lewicka et al^63^ isolated TiO_2_ and ZnO particles by several extraction processes and examined its shape, dimension, crystal phase, surface area, and elemental composition. These TiO_2_ and ZnO pigments exhibited two different sizes and forms. TiO_2_ pigment showed needle or nearly spherical shape and was smaller whereas ZnO pigment showed rod-like shape appearance and was much larger. The surface area of ZnO was found to be less than TiO_2_. The dimension of TiO_2_ was about 25 nm, and ZnO had short axis less than 40 nm and longer dimension greater than 100 nm. TEM helped to isolate and determine the primary pigment dimensions. This study compared the characters of the extracted sunscreen pigments with the commercially available nanomaterials, and two sources (T-Eco and Z-Cote) were identified which were similar to commercially obtained sunscreen materials. Elemental analysis showed that the sunscreen pigments contained aluminium and silicon and were similar to T-Eco and Z-Cote which had no additional inorganic elements. These aluminium and silicon were used as coating materials on TiO_2_ to reduce its UV photoactivity and restrict the reactive oxygen species production. This data would be helpful in studying the impact of engineered nanomaterials in sunscreen on the health of individuals and the environment.

Yenilmez et al^64^ formulated vitamin E incorporated chitosan (CS) microspheres for cosmetic purposes. This vitamin E incorporated CS microspheres were prepared and evaluated for various characteristics such as particle size, size distribution, zeta potential, and polymeric lattice structure. Vitamin E as such is not used widely in the cosmetic preparations because of its low stability, but vitamin E in CS microspheres proved to be relatively stable. When vitamin E was incorporated in CS microspheres, it showed enhanced cationic character of particles which resulted in enhanced topical penetration. The polymeric lattice of pure CS was in amorphous state and even after incorporation of vitamin E there was no change in the structure of particles. Incorporation efficiency of the vitamin E in CS microspheres was found to be 78.40 ± 2.41% (w/w). *In vitro* studies showed that vitamin E release from CS microsphere was 89.1% at the end of 6 hours. DPPH tests showed that this formulation had an antioxidant effect. *In vivo* efficiency tests of vitamin E formulation showed an increase in skin elasticity and moisture without a change in skin pH and sebum values, proving to be an effective anti-aging product.

Topical peptides have also been explored as a possible anti-aging strategy. Lim et al studied the enhanced penetration of Argireline^®^, a synthetic acetyl hexapeptide as a plausible compound for reducing the wrinkles associated with aging. Four peptide analogs which were chemically modified were investigated for its increased penetration ability. The wrinkle reducing effect of the peptides were also compared using human dental pulp stem cells. The peptides were dissolved in propylene glycol and water mixture to evaluate its permeation. The modified peptides were found to show an enhanced penetration and anti-wrinkling potential as compared to the parent compound.^65,66^

The effect of modulation of gene expression instigated by *Aframomum angustifolium* seed extract was evaluated as a possible anti-aging strategy by Talbourdet et al. The efficacy of the extracts was evaluated on normal human keratinocytes (NHKs) and human fibroblasts (NHFs). The expressions of antioxidant genes namely metallothionein 1, metallothionein 2, and thioredoxin were increased in NHKs, while expressions of glutathione peroxidase were increased in NHFs. On evaluating the efficacy of the skin care product containing the seed extract, a significant improvement in the homogeneity of the skin was observed. This suggests the potential of the seed extract as a possible gene expression modulator and its effect on the skin rejuvenation as a promising anti-wrinkling agent.^67^

Increasing age is often associated with a decrease in the stem cell production. They form the building blocks for regeneration. Presence of stem cells will help in repairing damaged areas by secretion of growth factors which can act as a potential strategy for skin therapeutics.^68^ Sych et al evaluated the potential for the use of fetal stem cells (FSCs) as an anti-aging strategy. Studies were conducted on 126 subjects which revealed that FSCs were found to be safe and efficacious for rejuvenation therapy.^69^ These results proved that stem cells could be a promising alternative to the currently available anti-aging products.

Fu et al. conducted a randomized trial on 196 women to evaluate the efficacy of a cosmetic product containing 5% niacinamide, peptides and 0.3 % retinyl propionate in comparison to the commercially available tretinoin as a potential anti-wrinkling agent. The results revealed a remarkable improvement in the fine lines and wrinkle appearance after 8 weeks when compared to that of prescription product, tretinoin.^70^

## Nanoparticles in anti-aging and sunscreens

Nanoparticles have been explored for its use in delivering a variety of cosmeceuticals. Conventional delivery systems have been replaced with novel nanocarriers like liposomes, niosomes, nanostructured lipid carriers, nanoemulsion, etc. Their stability, high entrapment efficiency, controlled release of the actives and enhanced skin penetration make it advantageous over the conventional counterparts. A plethora of literature is available on the use of nanoparticles as a delivery system for anti-aging and sunscreen effects. Heydari et al prepared nanoethosomal delivery system of gamma oryzanol for dermal delivery to prevent skin aging. The effect of the formulation on the skin wrinkle improvement was studied by dermoscopic and histological examination on healthy humans and rats. The results of histopathological studies revealed an improved antioxidant activity of the nanoethosomal formulation as compared to the plain gel. Positive results with respect to its photo protective effect were confirmed from dermoscopic studies.^71^

The photoprotective and antioxidant effects of naringenin were studied by formulating them into polymeric nanoparticles. The in vitro skin permeation studies revealed an increased deposition of naringenin in the skin rather than permeation into the deeper layers. The formulation also exhibited significant antioxidant effect with limited dermal toxicity. This suggests that the nanoparticulate formulation may behave as a potential antioxidant and sunscreen agent.^72^

Transferosomes loaded with epigallocatechin-3-gallate (EGCG) and hyaluronic acid were developed by Avadhani et al to enhance their sun protective activity along with their antioxidant and anti-aging effects. The developed nanoparticles showed promising free-radical scavenging and were able to suppress ROS and MDA levels when evaluated in human keratinocytes. An enhanced penetration and deposition of EGCG were found in the skin when given with hyaluronic acid.^73^ Shetty et al developed a novel sunscreen formulation containing morin which showed positive in vitro free radical scavenging properties. The nanoparticles were further incorporated into creams containing zinc oxide and nano titanium dioxide which showed optimum sun protection factor of ~40. In vivo studies on UV exposed rats revealed excellent antioxidant effects with least toxicity.^6^

To reduce irritation potential and to prolong the duration of action and increase the loading capacity of tretinoin (a vitamin A derivative), Ghate et al formulated nanostructured lipid carriers (NLCs). The NLCs were then incorporated into carbopol gel for the ease of application. On evaluating it’s in vitro release profile, a sustained release pattern was observed when compared to the marketed product. *In vivo* skin irritation studies showed minimal skin irritation with the developed formulations in comparison to marketed tretinoin gel.^74^

Ammar et al developed folic acid loaded nanostructured carriers to enable its controlled delivery for an anti-aging effect. The NLCs were fabricated using the hot high-pressure homogenization technique. The optimized nanoparticles were evaluated for their *in vitro* release, ex vivo skin permeation and antioxidant properties. About 95% of folic acid was found to be encapsulated within the lipid matrix. On determining its skin deposition ability, the results showed a promising accumulation within the skin revealing a depot effect. The developed formulations also showed a significant hydration and free radical scavenging potential suggesting the possibility of using this formulation as a potential anti-aging formulation.^75^

The protective effect of rice bran oil against UVB induced skin injury in mice when encapsulated within a lipid core nanocapsule was evaluated by Rigo et al. Nanocapsules were prepared using poly(ε-caprolactone) and span 60 which were later incorporated into hydrogels made of carbopol as gel base. The efficacy of the formulation was tested in UVB induced skin injury in mice. The in vivo results showed a promising protective and antioxidant effect of rice bran oil when encapsulated within nanocapsules as compared to rice bran oil alone. This technology suggests the critical role of nanoparticles as delivery carriers for the treatment of skin injuries triggered by UV radiations.^76^

## Patents on anti-aging and sunscreens

Due to the widespread market value of the anti-aging and sunscreen products, constant developments are being made to come out with new formulations for the same. Many patents have been filed by researchers, a few of which have been compiled in this section.

Gershon et al^77^ formulated sunscreen preparation containing zinc oxide (ZnO) particles. The particle size of ZnO was tailored in such a way that they were within the maximum threshold of 80 nm and a minimum threshold of 30 nm. ZnO has the ability to absorb UV radiation and protect the skin from further damage. These particles were further coated with the optical coating material and suspended in a suitable media which was selected based on its refractive index. Two or more media were combined together to achieve the desired refractive index between 1 and 2. Integration of the multiple zinc oxide particles was performed by dispersing it in the first medium and applying the second medium on the top. The multiple manipulated zinc oxide particles were optically coated which contains silicon dioxide. Each of these optically coated multiple zinc oxide particles undergoes aggregation and forms one or more clusters of size greater than 200 nm. In these clusters, the multiple zinc oxide particles create optical separation. The aggregates comprise one or more binding agent with one or more nanospheres.

Alexiades-Armenakas^78^ developed cosmetic preparation containing active ingredients to fight various signs of skin aging such as wrinkles, age spots, reduction in redness, acne, and rosacea. This single anti-aging skin care formulation contained 37 different microencapsulated ingredients which are protected from surrounding ingredients and also penetrates deeper strata of skin. The cream consisted of multiple ingredients along with a polymer base. The ingredients were selected based on their safety and efficacy, and belonged to the following categories, *viz,* DNA and cellular repair, anti-wrinkle, anti-redness and anti-pigmentation, antioxidant, anti UV damage, barrier repair, moisturizer, pro-collagen and an ingredient which prevents abnormal skin lesions. The ingredients having DNA repair and anti UV damage properties, procollagen were encapsulated in liposomes which contained soy lecithin to target epidermis. Cationic liposomes were chosen to ensure intercellular delivery of antioxidants including vitamin C and E for deeper penetration into the dermis layer. The liposomes consisted of an edge activator, sodium deoxycholate which helps in barrier repair and acts as an emollient.

Armand^79^ formulated an anti-aging cream containing equal mixture high molecular weight hyaluronic acid (HA) and low molecular weight hyaluronic acid oligosaccharides. Water-based hyaluronan was prepared to repair and prevent skin aging by preventing transepidermal water loss, damage to skin elastic fibers. This mixture of hyaluronan helps in the production of new collagen, skin keratinocytes, and extracellular matrix components. Low molecular weight hyaluronic acid oligosaccharides were prepared by hydrolyzing high molecular weight hyaluronic acid with testicular hyaluronidase, followed by its purification using ion-exchange resins. The obtained oligosaccharides were chemically deacetylated and mixed with an equal amount of high molecular weight hyaluronan in the final formulation. The penetrability of the formulation into the dermis was assessed using artificial skin and nude mice using isothiocyanatofluorescein coupled hyaluronan oligosaccharides.

Zahner^80^ formulated a topical sunscreen lotion containing ingredients in its natural state. This natural sunscreen with an aqueous phase had the composition as follows: melanin, green tea extract with a polyphenol, mineral pigments of titanium dioxide or zinc oxide. This all natural sunscreen lotion was formulated with the active ingredients such that it would provide desired SPF, protection of cell from free radicals and level of water resistance. The green tea polyphenols possess synergistic photoprotective effects on skin when combined with TiO_2_and ZnO and are effective in reducing erythema. Melanin present in the formulation is a superior free radical trap, and it is also capable of regenerating the neutralized polyphenols of green tea. The mineral pigments, TiO_2_ and ZnO, are said to be active UVA/UVB reflecting sunscreen ingredients which provide UVB protection of 75% for SPF 4, 88% for SPF 8 and 93% for SPF 15. These mineral sunscreens are left untreated such that this sunscreen composition remains in all natural state.

## Toxicity aspects

There are certain ingredients in the sunscreen that might cause hormonal changes in the body. Oxybenzone is an organic filter which absorbs UV radiations especially UVB radiation, and it is systemically absorbed and excreted in urine and feces.^81^ Oxybenzone is one such ingredient which affects the estrogenic levels in the body, and it has the highest rate of photoallergy among other UV filters.^45^ However, it was speculated that this effect could only be seen in individuals who would use the product continuously for a longer period for about 35-277 years.^54^ Nanoparticles can also cause local and systemic toxicity. Titanium dioxide and zinc oxide are reduced to nanosized particles so there is a great concern that these particles would penetrate the skin and produce UV induced free radicals.^81^ Continuous use of the sunscreen will prevent the exposure of the skin to UVB radiations thus there would be insufficient vitamin D synthesis in our body. Thus the individuals might not acquire the normal vitamin D levels which are required by the body. The dietary supplement would help to maintain the normal vitamin D levels in our body.^45^ Some chemical ingredients in sunscreen might cause edema, erythema, and irritation.^82^ To increase the patient compliance, several sunscreens have preservatives, fragrances, and other excipients which might induce allergic reactions in sensitive individuals. Patients with photodermatitis are likely to develop photo contact dermatitis to sunscreen.^83^ Sunscreens containing p-amino benzoic acid (PABA) help to protect from UVB rays and is said to be water resistant.^42^ But in vitro studies have shown that PABA interacts with DNA followed by UV radiations and might cause photocarcinogenesis.^48^ Hence a careful selection of sunscreen is vital to reap its benefits.

## Regulatory requirements/guidelines

As per the US Food and Drug Administration (FDA) laws, cosmetics products do not require pre-market approval from FDA. Nevertheless, these products are regulated by certain laws put forth by FDA.^84^ However, if these cosmetics contain any ingredients which may alter the body function, these are categorized as ‘drug’ as per Food, Drug and Cosmetic Act (FD&C). It is the responsibility of the company which manufactures these products to ensure the safety of such products.^85^

### Labeling information on sunscreen products

Some of the points which have to be displayed on the sunscreen label along with its explanation have been detailed below:

Broad spectrum: The label should specify if the sunscreen is a broad spectrum one. Sunscreens which are labeled as broad-spectrum protect our skin from both UVB and UVA rays. For a sunscreen to be broad spectrum it has to pass FDA broad spectrum test that measures the transmittance or absorbance of ultraviolet radiations across both UVA and UVB regions of the spectrum.^86^

**Figure 2:**
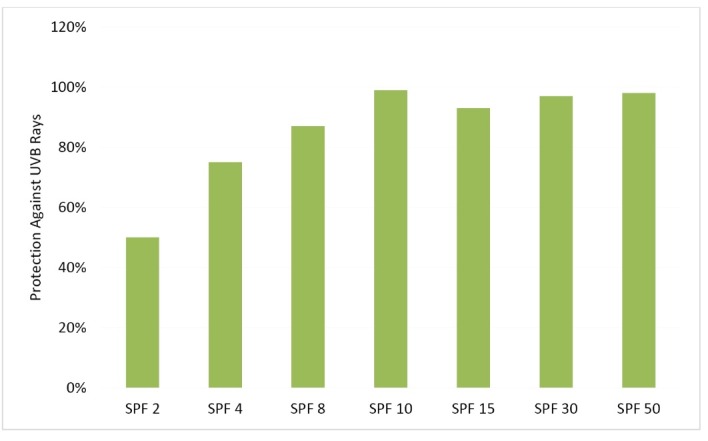
Protection rate of sunscreens with sun protection factor (SPF) from ultraviolet B (UVB) rays.

SPF: SPF is an important claim to be specified on the sunscreen products. SPF gives a measure of UVB rays involved in burning and not UVA rays involved in ageing.^21^ An SPF of 15 is expected to protect the skin from harmful UV rays for about 150 minutes.^87^ The levels of protection from different SPF rates are: SPF15 provides 93% of protection from UVB rays, SPF30 provides 97% of protection from UVB rays, and SPF50 provides 98% of protection from UVB rays (Figure 2).^88,89^

Thus we can conclude that SPF30 gives only 4% more protection and SPF50, about 5% more protection than SPF15.^88^ So it is a misapprehension that higher SPF can give more protection than that with a lower one.^90^

Water resistance: The sunscreen that claims to be water resistant should be reapplied for every 40 to 80 minutes which helps to provide protection while swimming and sweating.^91^

Shelf life: According to FDA, the shelf life of the sunscreen should be at least three years. The sunscreen products which does not have an expiry date mentioned on it clearly indicates that the shelf life of the product is three years.^86^ However, storage conditions can render the product unstable. Extreme temperatures also make the sunscreens less effective despite its expiration date.^51^

## Conclusion and future prospects

Although the sun is beneﬁcial and essential for life, exposing ourselves too much to sunlight might lead to detrimental health effects such as skin cancer. Studies and research have been conducted to introduce sunscreen in the form of pills for oral administration instead of reapplying the topical sunscreen repeatedly. Research and development are constantly underway to ensure that the sunscreen products are more effective and lessen the risk of adverse effects. Nanotechnology platforms have proved to be a major part of the cosmeceutical market owing to its better anti-aging and sunscreen potential which also renders a better skin deposition property and stability to the formulations. There has been ongoing research for the use of photosynthetic microorganisms in sunscreens especially cyanobacteria which has great potential in protecting our skin from damaging UV radiation and intense sunlight. Such biological compounds have many potential advantages over current synthetically derived sunscreens. The synthetic compounds are being gradually replaced with natural compounds as the new source of protective agents owing to their better efficacy and safety.

## Ethical Issues

Not applicable.

## Conflict of Interest

Authors declare no conflict of interest in this study.

## References

[1] Ganceviciene R, Liakou AI, Theodoridis A, Makrantonaki E, Zouboulis CC (2012). Skin anti-aging strategies. Dermatoendocrinol.

[2] Longo C, Casari A, Beretti F, Cesinaro AM, Pellacani G (2013). Skin aging: in vivo microscopic assessment of epidermal and dermal changes by means of confocal microscopy. J Am Acad Dermatol.

[3] Situm M, Buljan M, Cavka V, Bulat V, Krolo I, Mihic LL (2010). Skin changes in the elderly people--how strong is the influence of the UV radiation on skin aging?. Coll Antropol.

[4] Eklouh-Molinier C, Happillon T, Bouland N, Fichel C, Diebold MD, Angiboust JF (2015). Investigating the relationship between changes in collagen fiber orientation during skin aging and collagen/water interactions by polarized-FTIR microimaging. Analyst.

[5] Cadet J, Douki T, Pouget JP, Ravanat JL, Sauvaigo S (2001). Effects of UV and visible radiations on cellular DNA. Curr Probl Dermatol.

[6] Shetty PK, Venuvanka V, Jagani HV, Chethan GH, Ligade VS, Musmade PB (2015). Development and evaluation of sunscreen creams containing morin-encapsulated nanoparticles for enhanced UV radiation protection and antioxidant activity. Int J Nanomedicine.

[7] Makrantonaki E, Zouboulis CC (2007). Molecular mechanisms of skin aging: state of the art. Ann N Y Acad Sci.

[8] Poljsak B, Dahmane R (2012). Free radicals and extrinsic skin aging. Dermatol Res Pract.

[9] Kong BY, Sheu SL, Kundu RV (2015). Assessment of consumer knowledge of new sunscreen labels. JAMA Dermatol.

[10] Why Does Your Skin Age? http://dujs.dartmouth.edu/2013/01/why-does-your-skin-age/#.WnyBZ-hubIU. Accessed February 8, 2018.

[11] Li X (2015). Anti-aging cosmetics and its efficacy assessment methods. IOP Conf Ser Mater Sci Eng.

[12] Kohl E, Steinbauer J, Landthaler M, Szeimies RM (2011). Skin ageing. J Eur Acad Dermatol Venereol.

[13] Puizina-Ivic N (2008). Skin aging. Acta Dermatovenerol Alp Pannonica Adriat.

[14] Blasco MA (2005). Mice with bad ends: mouse models for the study of telomeres and telomerase in cancer and aging. EMBO J.

[15] Farage MA, Miller KW, Elsner P, Maibach HI (2008). Intrinsic and extrinsic factors in skin ageing: a review. Int J Cosmet Sci.

[16] Jenkins G (2002). Molecular mechanisms of skin ageing. Mech Ageing Dev.

[17] Shammas MA (2011). Telomeres, lifestyle, cancer, and aging. Curr Opin Clin Nutr Metab Care.

[18] Amaro-Ortiz A, Yan B, D’Orazio JA (2014). Ultraviolet radiation, aging and the skin: prevention of damage by topical cAMP manipulation. Molecules.

[19] Afanas’ev IB (2010). Signaling by reactive oxygen and nitrogen species in skin diseases. Curr Drug Metab.

[20] Imokawa G (2009). Mechanism of UVB-induced wrinkling of the skin: paracrine cytokine linkage between keratinocytes and fibroblasts leading to the stimulation of elastase. J Investig Dermatol Symp Proc.

[21] Mccullough JL, Kelly KM (2006). Prevention and treatment of skin aging. Ann N Y Acad Sci.

[22] Masaki H (2010). Role of antioxidants in the skin: anti-aging effects. J Dermatol Sci.

[23] Si H, Liu D (2014). Dietary antiaging phytochemicals and mechanisms associated with prolonged survival. J Nutr Biochem.

[24] Sniderman AD, Furberg CD (2008). Age as a modifiable risk factor for cardiovascular disease. Lancet.

[25] Camici GG, Shi Y, Cosentino F, Francia P, Luscher TF (2011). Anti-aging medicine: molecular basis for endothelial cell-targeted strategies - a mini-review. Gerontology.

[26] Ostan R, Bucci L, Capri M, Salvioli S, Scurti M, Pini E (2008). Immunosenescence and immunogenetics of human longevity. Neuroimmunomodulation.

[27] Makrantonaki E, Zouboulis CC, William  J (2007). Cunliffe Scientific Awards Characteristics and pathomechanisms of endogenously aged skin. Dermatology.

[28] Baumann L (2007). Skin ageing and its treatment. J Pathol.

[29] Sator PG, Schmidt JB, Sator MO, Huber JC, Honigsmann H (2001). The influence of hormone replacement therapy on skin ageing: a pilot study. Maturitas.

[30] Castelo-Branco C, Duran M, Gonzalez-Merlo J (1992). Skin collagen changes related to age and hormone replacement therapy. Maturitas.

[31] Rittie L, Kang S, Voorhees JJ, Fisher GJ (2008). Induction of collagen by estradiol: difference between sun-protected and photodamaged human skin in vivo. Arch Dermatol.

[32] Batra RS, Dover JS, Arndt KA (2005). Adverse event reporting for botulinum toxin type A. J Am Acad Dermatol.

[33] Cheng CM (2007). Cosmetic use of botulinum toxin type A in the elderly. Clin Interv Aging.

[34] Carruthers A, Carruthers J (2008). Botulinum toxin products overview. Skin Therapy Lett.

[35] Blanes-Mira C, Clemente J, Jodas G, Gil A, Fernandez-Ballester G, Ponsati B (2002). A synthetic hexapeptide (Argireline) with antiwrinkle activity. Int J Cosmet Sci.

[36] Sondh D, Parle A (2017). Anti-wrinkle agents-A way of regaining beauty. The Pharma Innovation Journal.

[37] Padamwar MN, Pawar AP, Daithankar AV, Mahadik KR (2005). Silk sericin as a moisturizer: an in vivo study. J Cosmet Dermatol.

[38] Huang CK, Miller TA (2007). The truth about over-the-counter topical anti-aging products: a comprehensive review. Aesthet Surg J.

[39] Tran D, Townley JP, Barnes TM, Greive KA (2015). An antiaging skin care system containing alpha hydroxy acids and vitamins improves the biomechanical parameters of facial skin. Clin Cosmet Investig Dermatol.

[40] Rigel DS (2002). Photoprotection: a 21st century perspective. Br J Dermatol.

[41] Forestier S (2008). Rationale for sunscreen development. J Am Acad Dermatol.

[42] Jallad KN (2017). Chemical characterization of sunscreens composition and its related potential adverse health effects. J Cosmet Dermatol.

[43] Nithya Shrikant. Why You Should Wear Sunscreen? - Top 11 Sunscreen Benefits. http://www.stylecraze.com/articles/sunscreen-why-use/#gref. Accessed February 9, 2018.

[44] Latha MS, Martis J, Shobha V, Sham Shinde R, Bangera S, Krishnankutty B (2013). Sunscreening agents: a review. J Clin Aesthet Dermatol.

[45] Jansen R, Osterwalder U, Wang SQ, Burnett M, Lim HW (2013). Photoprotection: part II Sunscreen: development, efficacy, and controversies. J Am Acad Dermatol.

[46] Moloney FJ, Collins S, Murphy GM (2002). Sunscreens: safety, efficacy and appropriate use. Am J Clin Dermatol.

[47] Antoniou C, Kosmadaki MG, Stratigos AJ, Katsambas AD (2008). Sunscreens--what’s important to know. J Eur Acad Dermatol Venereol.

[48] Gasparro FP, Mitchnick M, Nash JF (1998). A review of sunscreen safety and efficacy. Photochem Photobiol.

[49] Kaimal S, Abraham A (2011). Sunscreens. Indian J Dermatol Venereol Leprol.

[50] Serpone N, Dondi D, Albini A (2007). Inorganic and organic UV filters: Their role and efficacy in sunscreens and suncare products. Inorganica Chim Acta.

[51] Fayed L. Should You Use Sunscreen or Sunblock??Is There a Difference Between Them? http://www.wkiki.com/should-you-use-sunscreen-or-sunblock-is-there-a-difference-between-them-by-lisa-fayed-reviewed-doru-paul-md/. Accessed February 10, 2018.

[52] Haywood R, Wardman P, Sanders R, Linge C (2003). Sunscreens inadequately protect against ultraviolet-A-induced free radicals in skin: implications for skin aging and melanoma?. J Invest Dermatol.

[53] Chen L, Hu JY, Wang SQ (2012). The role of antioxidants in photoprotection: a critical review. J Am Acad Dermatol.

[54] Mancuso JB, Maruthi R, Wang SQ, Lim HW (2017). Sunscreens: An Update. Am J Clin Dermatol.

[55] SPF 30 Mineral Sunscreen Fluid for Face:. Clinique website. https://www.clinique.com/product/1661/40649/sun/sun-protection/spf-30-mineral-sunscreen-fluid-for-face. Accessed April 13, 2019.

[56] Activated Sun Protector Sunscreen for Face and Body SPF50. https://www.kiehlstimes.com.my/product/activated-sun-protector-sunscreen-for-face-and-body-spf50/. Accessed April 13, 2019.

[57] The best anti-aging cream: Sunscreen. https://www.laroche-posay.co.uk/the-best-anti-aging-cream-sunscreen. Accessed April 13, 2019.

[58] What’s the best anti-aging cream - NIVEA. https://www.nivea.com.au/advice/face-care/anti-ageing/tips-and-treatments/what-is-the-best-anti-aging-cream. Accessed April 13, 2019.

[59] Clarins sun wrinkle control cream SPF15 review. https://makeupandbeauty.com/clarins-sun-wrinkle-control-cream-review/. Accessed April 13, 2019.

[60] Ultrasun SPF30 Face Anti-Ageing Formula 50ml. Victoria Health website. https://www.victoriahealth.com/product/Ultrasun-Face-SPF30-Anti-Ageing-Formula/9294. Accessed April 13, 2019.

[61] Alpha-H Protection Plus Daily SPF 50+ Broad Spectrum Cream Review. Makeupandbeauty website. https://makeupandbeauty.com/alpha-h-protection-plus-daily-spf-50-broad-spectrum-cream-review/. Accessed April 13, 2019.

[62] Tinted Face Sunscreen - SPF 30. Frezyderm website. https://www.frezyderm.co.uk/sun-care/tinted-face-sunscreens/sun-screen-color-velvet-face-spf-30/. Accessed April 13, 2019.

[63] Lewicka ZA, Benedetto AF, Benoit DN, Yu WW, Fortner JD, Colvin VL (2011). The structure, composition, and dimensions of TiO2 and ZnO nanomaterials in commercial sunscreens. J Nanopart Res.

[64] Yenilmez E, Başaran E, Yazan Y (2011). Release characteristics of vitamin E incorporated chitosan microspheres and in vitro–in vivo evaluation for topical application. Carbohydr Polym.

[65] Gorouhi F, Maibach HI (2009). Role of topical peptides in preventing or treating aged skin. Int J Cosmet Sci.

[66] Lim SH, Sun Y, Thiruvallur Madanagopal T, Rosa V, Kang L (2018). Enhanced Skin Permeation of Anti-wrinkle Peptides via Molecular Modification. Sci Rep.

[67] Talbourdet S, Sadick NS, Lazou K, Bonnet-Duquennoy M, Kurfurst R, Neveu M (2007). Modulation of gene expression as a new skin anti-aging strategy. J Drugs Dermatol.

[68] Ullah M, Sun Z (2018). Stem cells and anti-aging genes: double-edged sword-do the same job of life extension. Stem Cell Res Ther.

[69] Sych N, Klunnyk M, Matiyashchuk I, Demchuk M, Ivankova O, Sinelnyk A (2017). Fetal stem cells use as antiaging and rejuvenation strategies. J Cosmo Trichol.

[70] Fu JJ, Hillebrand GG, Raleigh P, Li J, Marmor MJ, Bertucci V (2010). A randomized, controlled comparative study of the wrinkle reduction benefits of a cosmetic niacinamide/peptide/retinyl propionate product regimen vs a prescription 002% tretinoin product regimen. Br J Dermatol.

[71] Heydari S, Ghanbarzadeh S, Anoush B, Ranjkesh M, Javadzadeh Y, Kouhsoltani M (2017). Nanoethosomal formulation of gammaoryzanol for skin-aging protection and wrinkle improvement: a histopathological study. Drug Dev Ind Pharm.

[72] Joshi H, Hegde AR, Shetty PK, Gollavilli H, Managuli RS, Kalthur G (2018). Sunscreen creams containing naringenin nanoparticles: Formulation development and in vitro and in vivo evaluations. Photodermatol Photoimmunol Photomed.

[73] Avadhani KS, Manikkath J, Tiwari M, Chandrasekhar M, Godavarthi A, Vidya SM (2017). Skin delivery of epigallocatechin-3-gallate (EGCG) and hyaluronic acid loaded nano-transfersomes for antioxidant and anti-aging effects in UV radiation induced skin damage. Drug Deliv.

[74] Ghate VM, Lewis SA, Prabhu P, Dubey A, Patel N (2016). Nanostructured lipid carriers for the topical delivery of tretinoin. Eur J Pharm Biopharm.

[75] Ammar HO, Ghorab MM, Mostafa DM, Ibrahim ES (2016). Folic acid loaded lipid nanocarriers with promoted skin antiaging and antioxidant efficacy. J Drug Deliv Sci Technol.

[76] Rigo LA, da Silva CR, de Oliveira SM, Cabreira TN, 
de Bona da Silva
 
C
, Ferreira J (2015). Nanoencapsulation of rice bran oil increases its protective effects against UVB radiation-induced skin injury in mice. Eur J Pharm Biopharm.

[77] Gershon TS, Li N, Sadana D, Todorov TK. Controlling zinc oxide particle size for sunscreen applications. US20170065505 A1, 2016.

[78] Alexiades-Armenakas M. Multi-active microtargeted anti-aging skin care cream polymer technology. US8529925 B2, 2012.

[79] Armand G. Topical anti-wrinkle and anti-aging moisturizing cream. US 20100098794, 2010.

[80] Zahner P. All natural sunscreen lotion. US20050042186 A1, 2003.

[81] Mancebo SE, Hu JY, Wang SQ (2014). Sunscreens: a review of health benefits, regulations, and controversies. Dermatol Clin.

[82] Kale S, Gaikwad M, Bhandare S (2011). Determination and comparison of in vitro SPF of topical formulation containing Lutein ester from Tagetes erecta L Flowers, Moringa oleifera Lam seed oil and Moringa oleifera Lam seed oil containing Lutein ester. Int J Res Pharm Biomed Sci.

[83] Heurung AR, Raju SI, Warshaw EM (2014). Adverse reactions to sunscreen agents: epidemiology, responsible irritants and allergens, clinical characteristics, and management. Dermatitis.

[84] Nutrition C for FS and A. Cosmetics Q&A: Why are cosmetics not FDA-approved? https://www.fda.gov/Cosmetics/ResourcesForYou/Consumers/ucm135709.htm. Accessed February 10, 2018.

[85] Nutrition C for FS and A. FDA Authority Over Cosmetics: How Cosmetics are not FDA-Approved, but are FDA-Regulated. https://www.fda.gov/Cosmetics/GuidanceRegulation/LawsRegulations/ucm074162.htm. Accessed February 10, 2018.

[86] Research C for DE and. Sunscreen: How to Help Protect Your Skin from the Sun. https://www.fda.gov/drugs/understanding-over-counter-medicines/sunscreen-how-help-protect-your-skin-sun. Accessed February 10, 2018.

[87] Hanrahan JR (2012). Sunscreens. Aust Prescr.

[88] Draelos ZD (2011). The multifunctional value of sunscreen-containing cosmetics. Skin Therapy Lett.

[89] Sun Protection Factor (SPF) and Sunscreen. https://www.verywellhealth.com/spf-sun-protection-factor-and-sunscreen-2634104. Accessed April 12, 2019.

[90] Hughes MC, Williams GM, Baker P, Green AC (2013). Sunscreen and prevention of skin aging: a randomized trial. Ann Intern Med.

[91] Hexsel CL, Bangert SD, Hebert AA, Lim HW (2008). Current sunscreen issues: 2007 Food and Drug Administration sunscreen labelling recommendations and combination sunscreen/insect repellent products. J Am Acad Dermatol.

